# Female and preserved platelet count subgroups of myelodysplastic syndrome patients benefit from standard‐dose azacitidine

**DOI:** 10.1002/cnr2.1938

**Published:** 2023-11-28

**Authors:** Shinichi Ogawa, Tatsuhiro Sakamoto, Ryota Matsuoka, Kantaro Ishitsuka, Yasuko Ogino, Ayano Sootome, Kenichi Makishima, Chikashi Yoshida, Yufu Ito, Seiichi Shimizu, Takuya Suyama, Atsushi Shinagawa, Takayoshi Ito, Naoshi Obara, Manabu Kusakabe, Mamiko Sakata‐Yanagimoto, Yasushi Miyazaki, Yasuhito Nannya, Shigeru Chiba

**Affiliations:** ^1^ Division of Hematology JA Toride General Medical Center Toride, Ibaraki Japan; ^2^ Department of Hematology, Faculty of Medicine University of Tsukuba Tsukuba, Ibaraki Japan; ^3^ Department of Pathology, Faculty of Medicine University of Tsukuba Tsukuba, Ibaraki Japan; ^4^ Graduate School of Comprehensive Human Sciences University of Tsukuba Tsukuba, Ibaraki Japan; ^5^ Division of Hematology National Hospital Organization Mito Medical Center Mito, Ibaraki Japan; ^6^ Division of Hematology Tsuchiura Kyoudou General Hospital Tsuchiura, Ibaraki Japan; ^7^ Division of Hematology Hitachi General Hospital Hitachi, Ibaraki Japan; ^8^ Department of Hematology Atomic Bomb Disease Institute, Nagasaki University Nagasaki Japan; ^9^ Department of Hematology Institute of Medical Science, University of Tokyo Tokyo Japan

**Keywords:** azacitidine, dose, myelodysplastic syndrome, platelet counts, sex

## Abstract

**Background:**

Hypomethylating agents, including azacytidine (AZA), are standard therapeutics for patients with high‐risk myelodysplastic syndromes (MDS), a group of myeloid neoplasms. However, treatment schedules are not unified in real‐world practice; in addition to the standard 7‐day (standard‐dose) schedule, shortened (reduced‐dose) schedules are also used.

**Aims:**

The aim of this study was to discover the patient group(s) which show differential efficacy between standard‐and reduced‐dose AZA to MDS.

**Methods and Results:**

The outcome of different AZA doses in a cohort of 151 MDS patients were retrospectively analyzed. Overall survival (OS) was not significantly different between standard‐ and reduced‐dose AZA groups by multivariate analysis. However, an interaction was found between either the sex (female vs. male), the platelet counts (< 40 × 10^3^/μl vs. ≥ 40 × 10^3^/μl), or the karyotype risk (< poor vs. ≥ poor) and standard‐dose AZA for longer OS. Subgroup analyses revealed better OS with standard‐ over reduced‐dose AZA in female patients (HR, 0.27 [95% CI, 0.090‐0.79]; *p* = 0.011), and those with platelet counts ≥ 40 × 10^3^/μl (HR, 0.51 [95% CI, 0.26‐0.99]; *p* = 0.041). The union of female and preserved platelet count subgroups also benefited from standard‐dose AZA. With this as a test cohort, we next analyzed patients registered in the JALSG MDS212 study, for whom 7‐day and 5‐day AZA treatment strategies were prospectively compared, as a validation cohort (*N* = 172). That cohort showed the same tendency as the retrospective results.

**Conclusion:**

We identified the union of female and preserved platelet count subgroups which benefited from standard‐dose AZA, imparting crucial information to physicians planning treatment regimens in MDS patients.

## 1 INTRODUCTION

Myelodysplastic syndromes (MDS) are a group of chronic myeloid neoplasms characterized by pancytopenia, dysplasia, and predisposition to acute myeloid leukemia (AML).^1^, ^2^ The mainstay of therapy includes hypomethylating agents (HMAs), including azacitidine (AZA), and hematopoietic stem cell transplantation (HSCT) if eligible.^3^, ^4^, ^5^, ^6^ In the standard protocol, AZA is given at 75 mg/m^2^ per day for 7 consecutive days every 28 days based on a Phase III study demonstrating prolonged overall survival (OS).^5^ High‐risk MDS patients are also reported to respond to shortened schedules (reduced‐doses) of AZA although OS benefit differences between standard‐ and reduced‐doses are controversial.^7^, ^8^, ^9^, ^10^, ^11^ Based on these reports, the 5‐day protocol is often clinically used because of convenience and better tolerability.^12^ If the standard protocol is superior to the reduced protocol, the patients who receive the reduced protocol may lengthen their OS by changing their administration protocol. On the other hand, if the reduced protocol is equal to the standard protocol, it needs to be considered whether the standard protocol is reconsidered to lighten the adverse effects on patients and reduce economic burden to both patients and the society. Therefore, it is important to exhaustively compare the efficacy and difference between the protocols. Because AZA is a backbone of new combinatorial therapies for MDS and AML with venetoclax, magrolimab, APR246, and so on,^13^, ^14^, ^15^, ^16^, ^17^ detailed data between the standard and the reduced doses may influence clinical studies and resulting new therapeutic regimens. Although a Phase III clinical trial was conducted to prove the superiority of the 7‐day over the 5‐day protocol, it was never completed and statistically significant OS differences between the 7‐ and 5‐day protocols were not proven.^18^

We had a community‐based information that reduced‐dose AZA is prescribed to a significant proportion of MDS patients in Ibaraki Prefecture in Japan. Thus, we conducted a multicenter retrospective study to disclose real‐world dosing schedules and investigate any potential differences in OS between patients receiving AZA at standard‐ or reduced‐doses. Furthermore, we intended to identify subcohorts in which AZA dose delineated OS. To define such subcohorts, interaction analyses between cumulative AZA dose and each clinical parameter were performed as screening before subgroup analyses were performed for selected parameters. Sex and platelet count were each related to AZA dose dependency.

After validation with a prospective cohort registered in the JALSG MDS212 study,^18^ it was suggested that the standard‐ or near standard‐dose of AZA, in comparison with the reduced‐dose, improved OS in female patients and those with preserved platelet counts.

## 2 METHODS

### 2.1 Patients and inclusion criteria for clinical analyses

One‐hundred and eighty‐six patients were enrolled, all diagnosed with MDS according to either the FAB^19^ or the WHO 2016 criteria,^1^ and treated by AZA from March 2011 to May 2019 at 5 hospitals in Ibaraki Prefecture. Two patients with a history of HSCT before AZA administration and one with a shortage of clinical data were removed (Table S1).

To investigate the influence of differences in the dose of AZA on hematological improvement (HI) and OS, we further removed 32 patients who died sooner than day 112 after the commencement of AZA. Then, resulting 151 patients who survived for 112 (28 days × 4 courses) days or longer (survivor112) were determined as a main target of our analysis. This was because we planned to exclude the short survivors dying sooner than day 112 based on our understanding represented by the following reports. First, the median number of courses required for the initial response was three, and 90% of responses were seen by 6 courses in MDS.^20^ Second, AZA should be continued for at least 4–6 courses to judge whether the patients respond to AZA or not in AML patients.^21^ Therefore, we collected the cumulative AZA dose at day 112, as well as data on the total number of AZA treatment course and the mean period of AZA administration in each course (6 days or shorter, or longer than 6 days). This retrospective study was approved by the institutional review board in each hospital. This retrospective study was based on the medical records. Obeying the approval of each institutional review board, we performed opt‐out in each hospital instead of written informed consent.

### 2.2 Definitions of hematological improvement, survival, and cumulative AZA dose

Hematological improvement (HI) to AZA was defined according to the revised IWG 2019 hematological response criteria.^22^ OS was defined as the time from the day of the first administration of AZA to the day of death caused by any reasons. Living patients were censored at the last contact and those patients receiving stem cell transplantation were censored at the day of the stem cell infusion. Cumulative doses of AZA (mg/m^2^) in the first 4 courses were calculated by dividing the sum of AZA given on or before day 112 by the body surface area at the first administration of AZA. If AZA was administered at 75 mg/m^2^/day for 5 days and body surface area was unchanged, the cumulative AZA dose was considered to be 1500 mg/m^2^. Based on this calculation, cumulative AZA doses equal to or less than 1500 mg/m^2^ were defined as reduced‐dose while over 1500 mg/m^2^ was defined as the standard‐dose.

### 2.3 Statistics

Fisher's exact test was used for univariate analyses of binary variables for response to AZA while the Mann–Whitney U test was used for univariate analysis of continuous variables. Logistic regression modeling was used for multivariate analyses of binary variables for response to AZA. OS was evaluated using the Kaplan–Meier method. The log‐rank test was used to compare the survival curves between the patient groups of interest. The Cox proportional hazard model was used to estimate hazard ratios (HR) and 95% confidence intervals (95% CI) of HR in univariate and multivariate analyses of OS. In multivariate analyses of hematological improvement rate and OS, age (<75 vs. ≥75), sex, karyotype risk defined by the revised international prognostic scoring system criteria (IPSS‐R) (<poor vs. ≥poor),^23^ bone marrow blast percentage (<10% vs. ≥10%), neutrophile counts (<800/μl vs. ≥800/μl), hemoglobin levels (<8 g/dL vs. ≥8 g/dL), platelet counts (<40 × 10^3^/μl vs. ≥40 × 10^3^/μl), and cumulative AZA doses (reduced‐dose vs. standard‐dose) were included as explanatory variables, irrespective of *p* values. Factors with *p* values <.05 were additionally included in explanatory variables. To obtain the propensity score (PS), the probability to receive the standard‐dose was calculated using a logistic regression model in which explanatory variables were age, sex, bone marrow blast percentage, WHO 2016 diagnosis, karyotype‐risk defined by IPSS‐R, with or without transplantation, neutrophile counts (Neu; /μL), hemoglobin levels (Hb; g/dL), and platelet counts (Plt; × 10^4^/μL) at the first administration of AZA. The PS matching was performed using 1:1 caliper matching (caliper 0.2). Statistical analyses were performed using EZR.^24^

## 3 RESULTS

### 3.1 Patient characteristics

Characteristics of the 183 patients are shown in Table S1. The median age was 72 years (range, 29–90) with a male/female ratio of 2.05. Myelodysplastic syndrome with excess blasts 1 (MDS‐EB1) and MDS‐EB2 were the most prevalent (62.8%), followed by MDS with multiple lineage dysplasia (MDS‐MLD; 19.1%) and AML with myelodysplasia‐related changes (AML‐MRC; 10.4%), according to the WHO 2016 criteria. All AML‐MRC cases corresponded to refractory anemia with excess blasts (RAEB) in transformation (RAEB‐t) according to the FAB classification. Based on IPSS‐R, 71.1% were judged to have high‐ or very high‐risk prognosis. The median number of AZA courses was 6 (range, 1–61).

Of Survivors112, the standard‐ and reduced‐doses were given to 91 and 60 patients, respectively (Table 1). Median cumulative AZA doses at day 112 were 2074 mg/m^2^ (10–90 percentile, 1575–2100 mg/m^2^) and 1232 mg/m^2^ (853–1500 mg/m^2^) in the standard‐ and reduced‐dose groups, respectively (Figure S1). The median Hb concentrations at the first administration of AZA were significantly higher in the standard‐dose group than the reduced‐dose group (8.6 g/dL vs. 7.8 g/dL, *p* = .04, Table 1), which potentially influenced the choice of the AZA dose. All other factors, including age, sex, diagnosis, IPSS‐R‐risk, karyotype‐risk, Neu and Plt at the first administration of AZA, and bone marrow blast percentage at diagnosis or within 3 months before AZA start, were not significantly different between the two groups.

**TABLE 1:** Patient characteristics divided by cumulative dose of AZA at day 112.

		Cumultive dose of AZA at day 112		
	All patients (%)	≤1500 mg/m^2^ (%)	>1500 mg/m^2^ (%)	*p* value
*N*	151	60	91	
Age, median [range]	72 [29, 90]	74 [29, 90]	72 [42, 86]	.47
Sex				1
Male	102 (67.5)	41 (68.3)	61 (67.0)	
Female	49 (32.5)	19 (31.7)	30 (33.0)	
WHO 2016 criteria				.30
MDS‐SLD	4 (2.6)	1 (1.7)	3 (3.3)	
MDS‐MLD	27 (17.9)	10 (16.7)	17 (18.7)	
MDS‐EB1	50 (33.1)	25 (41.7)	25 (27.5)	
MDS‐EB2	46 (30.4)	15 (25.0)	31 (34.1)	
AML‐MRC	17 (11.3)	6 (10.0)	11 (12.1)	
MDS with isolated del(5q)	1 (0.7)	1 (1.7)	0 (0.0)	
MDS‐RS	2 (1.3)	0 (0.0)	2 (2.2)	
CMML	1 (0.7)	0 (0.0)	1 (1.1)	
tMN	2 (1.3)	2 (3.3)	0 (0.0)	
MDS‐U	1 (0.7)	0 (0.0)	1 (1.1)	
IPSS‐R risk group				.30
Very low	2 (1.3)	0 (0.0)	2 (2.2)	
Low	16 (10.6)	7 (11.7)	9 (9.9)	
Intermediate	26 (17.2)	6 (10.0)	20 (22.0)	
High	50 (33.1)	22 (36.7)	28 (30.8)	
Very high	5 (37.1)	25 (41.7)	31 (34.1)	
NA	1 (0.7)	0 (0.0)	1 (1.1)	
IPSS‐R karyotype group				.17
Very good	3 (2.0)	0 (0.0)	3 (3.3)	
Good	61 (40.4)	18 (30.0)	43 (47.3)	
Intermediate	33 (21.9)	15 (25.0)	18 (19.8)	
Poor	11 (7.3)	6 (10.0)	5 (5.5)	
Very poor	39 (25.8)	19 (31.7)	20 (22.0)	
NA	4 (2.6)	2 (3.3)	2 (2.2)	
Transplantation				1
No	137 (90.7)	55 (91.7)	82 (90.1)	
Yes	14 (9.3)	5 (8.3)	9 (9.9)	
Bone marrow blast %, median [range]	7.6 [0.0, 29.8]	7.0 [0.4, 26.5]	8.4 [0.0, 29.8]	.56
Neutrophile count (/μL), median [range]	888 [47, 22 243]	904 [110, 22 243]	880 [47, 19 757]	.76
Hemoglobin (g/dL), median [range]	8.1 [2.4, 12.9]	7.8 [4.2, 12.9]	8.6 [2.4, 12.9]	.040
Platelet count (× 10^3^/μL), median [range]	63 [5, 629]	64 [10, 629]	62 [5, 364]	.32
Hematological improvement rate, % (95% CI)	54.1 (45.7–62.4)	36.2 (24.0–49.9)	65.9 (55.0–75.7)	.00064
Median follow up time, days (95% CI)	427 (364–449)	349.5 (263–428)	445 (400–522)	.35
Median survival time, days (95% CI)	509 (445–640)	427 (321–584)	623 (482–850)	.010
OS at 1 year, % (95% CI)	72.7 (64.2–79.6)	59.1 (44.6–71.0)	82.0 (71.4–88.9)	
OS at 2 year, % (95% CI)	35.9 (26.8–45.0)	24.3 (13.3–37.2)	44.7 (31.9–56.7)	

*Note*: Patients were included whoose overall survival was 112 days or longer.

Abbreviations: 95% CI, 95% confidence interval; AML‐MRC, acute myeloid leukemia with myelodysplasia‐related changes; CMML, chronic myelomonocytic leukemia; IPSS‐R, revised international prognostic scoring system; MDS‐EB1, myelodysplastic syndrome with excess blasts 1; MDS‐EB2, myelodysplastic syndrome with excess blasts 2; MDS‐MLD, myelodysplastic syndrome with multilineage dysplasia; MDS‐SLD, myelodysplastic syndrome with single lineage dysplasia; MDS‐RS, myelodysplastic syndrome with ring sideroblasts; MDS‐U, myelodysplastic syndrome, unclassifieable; OS, overall survival; NA, not available; tMN, therapy related myeloid neoplasms.

### 3.2 Hematological improvement

The hematological improvement (HI) rate in any parameter by AZA was 54.1% in Survivors112 (95% CI, 45.7%–62.4%) (Table 1). In univariate analyses, cumulative AZA dose and sex significantly affected the HI rate; these rates were greater with regard to standard‐dose and male sex (Tables 1 and S2). All other factors, such as age (<75 or ≥75), bone marrow blast percentage (<10% or ≥10%), or IPSS‐R‐risk (< high or ≥high), karyotype‐risk (< poor or ≥poor), Neu, Hb, and Plt, did not significantly affect the HI rate. In our multivariate analysis, sex and the cumulative AZA dose were again the significant parameter affecting the HI rate (Table S2). Response to AZA based on bone marrow evaluations could not be investigated because these data at the appropriate time points after AZA initiation were missing in a substantial number of patients.

### 3.3 Survival in the entire Survivors112 cohort

Median survival time (MST) was 509 days (95% CI, 445–640 days), while 1‐year OS was 72.7% (95% CI, 64.2%–79.6%) in the Survivors112 cohort (Table 1). MST and 1‐year OS in the whole cohort (183 patients) were described in Table S1. In univariate analyses, high or very high IPSS‐R (HR, 1.85 [95% CI, 1.10–3.12]; *p* = .019), poor or very poor karyotype‐risk (HR, 3.29 [95% CI, 2.04–5.31]; *p* = 2.5 × 10^−7^), no HI by AZA (HR, 2.21 [95% CI, 1.43–3.42]; *p* = 2.5 × 10^−4^), Hb <8 g/dL (HR, 0.62 [95% CI, 0.40–0.95]; *p* = .025), and the reduced‐dose (HR, 0.58 [95% CI, 0.38–0.88]; *p* = .010) were significant factors for poor prognosis (Table S3 and Figure S2). In the multivariate Cox proportional hazards model, poor or very poor karyotype‐risk (HR, 3.14 [95% CI, 1.88–5.23]; *p* = 1.2 × 10^−5^) and no HI (HR, 1.89 [95% CI, 1.13–3.18]; *p* = .016) significantly shortened the OS. The cumulative AZA dose was not an independent significant prognostic factor (Table S3).

Consequently, poor or very poor karyotype risk and no HI from AZA were negative prognostic factors in both univariate and multivariate analyses for the entire Survivors112 cohort, similarly to previously verified reports.

### 3.4 Survival in the subcohorts

**FIGURE 1:**
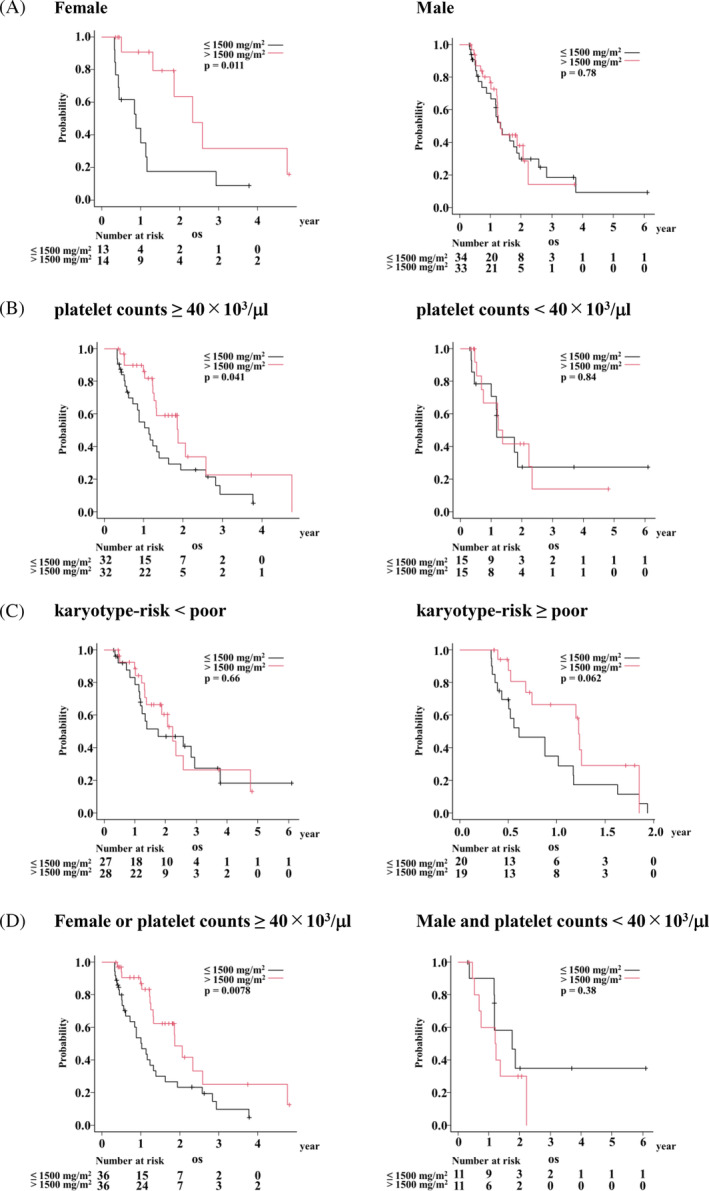
Subgroup analyses by sex, platelet counts, or karyotype in the propensity score‐matched analysis. Female and male (A), platelet counts <40 × 10^3^/μl and ≥40 × 10^3^/μl (B), and karyotype‐risk <poor and ≥poor (C), and the union of female and platelet counts ≥40 × 10^3^/μl, and patients other than the union (D).

Because the univariate analysis showed OS differences between the AZA doses, we hypothesized that the benefit of the standard‐dose AZA would be clearer if confounding factors were excluded. To remove such confounding factors and delineate subcohorts in which the standard‐dose AZA prolonged OS than the reduced‐dose AZA, we selected 94 patients by propensity score matching from Survivors112 (Table S4, Figure S3) and performed interaction analyses between each clinical parameter and cumulative AZA dose. We picked up any interactions providing *p* values of interaction less than .30. Sex (female vs. male), platelet counts (<40 × 10^3^/μl vs. ≥40 × 10^3^/μl), and karyotype risk (<poor vs. ≥poor) matched the criteria and were selected as candidates for the subcohorts (Table 2). Then, univariate analyses were performed in each subcohort to investigate whether cumulative AZA dose influenced OS. The standard‐dose significantly prolonged OS in the female (HR, 0.27 [95% CI, 0.090–0.79]; *p* = .011) and platelet counts ≥40 × 10^3^/μl (HR, 0.51 [95% CI, 0.26–0.99]; *p* = .041) subcohorts (Table 3 and Figure 1). In the karyotype‐risk ≥ poor subcohort, there was a tendency that the standard‐dose prolonged OS but without statistical significance (HR, 0.48 [95% CI, 0.22–1.07]; *p* = .062). The union of the female and platelet counts ≥40 × 10^3^/μl subcohorts, in other words, the patients other than the male with platelet counts <40 × 10^3^/μl, was delineated as the subcohort in which the standard‐dose AZA improved OS than the reduced‐dose AZA (HR, 0.43 [95% CI, 0.23–0.82]; *p* = .0078) (Table 3 and Figure 1).

**TABLE 2:** The *p* values of interaction with cumulative AZA dose in the propensity score‐matched analysis.

Age (<75 vs. ≥75)	.91
Sex (female vs. male)	.040
Bone marrow blast percentage (<10% vs. ≥10%)	.51
Neutrophile count (<800/μL vs. ≥800/μL)	.83
Hemoglobin (<8 g/dL vs. ≥8 g/dL)	.98
Platelet counts (<40 × 10^3^/μL vs. ≥40 × 10^3^/μL)	.28
Karyotype‐risk (<poor vs. ≥poor)	.25

**TABLE 3:** Subgroup analyses of overall survival in the propensity score‐matched analysis.

	Cumulative dose of AZA at day 112	*N*	Median survival time (95% CI) (Day)	HR (95% CI)	*p* value
Female					.011
	≤1500 mg/m^2^	13	321 (129–422)		
	>1500 mg/m^2^	14	850 (479–1737)	0.27 (0.090–0.79)	
Male					.78
	≤1500 mg/m^2^	34	484 (371–707)		
	>1500 mg/m^2^	33	482 (438–812)	0.91 (0.48–1.72)	
Platelet count <40 × 10^3^/μL					.84
	≤1500 mg/m^2^	15	429 (164–NA)		
	>1500 mg/m^2^	15	473.5 (192–850)	0.91 (0.36–2.30)	
Platelet count ≥40 × 10^3^/μL					.041
	≤1500 mg/m^2^	32	412 (222–594)		
	>1500 mg/m^2^	32	682 (458–940)	0.51 (0.26–0.99)	
Karyotype‐risk < poor					.66
	≤1500 mg/m^2^	27	640 (422–1072)		
	>1500 mg/m^2^	28	812 (482–1737)	0.85 (0.41–1.76)	
Karyotype‐risk ≥ poor					.062
	≤1500 mg/m^2^	20	222 (141–427)		
	>1500 mg/m^2^	19	448 (248–NA)	0.48 (0.22–1.07)	
Female or Platelet counts ≥40 × 10^3^/μL					.0078
	≤1500 mg/m^2^	36	371 (222–505)		
	>1500 mg/m^2^	36	682 (458–940)	0.43 (0.23–0.82)	
Male and Platelet counts <40 × 10^3^/μL					.38
	≤1500 mg/m^2^	11	640 (132–NA)		
	>1500 mg/m^2^	11	443 (167–NA)	1.60 (0.55–4.64)	

Abbreviations: 95% CI, 95% confidence interval; HR, hazard ratio; NA, not available.

**FIGURE 2:**
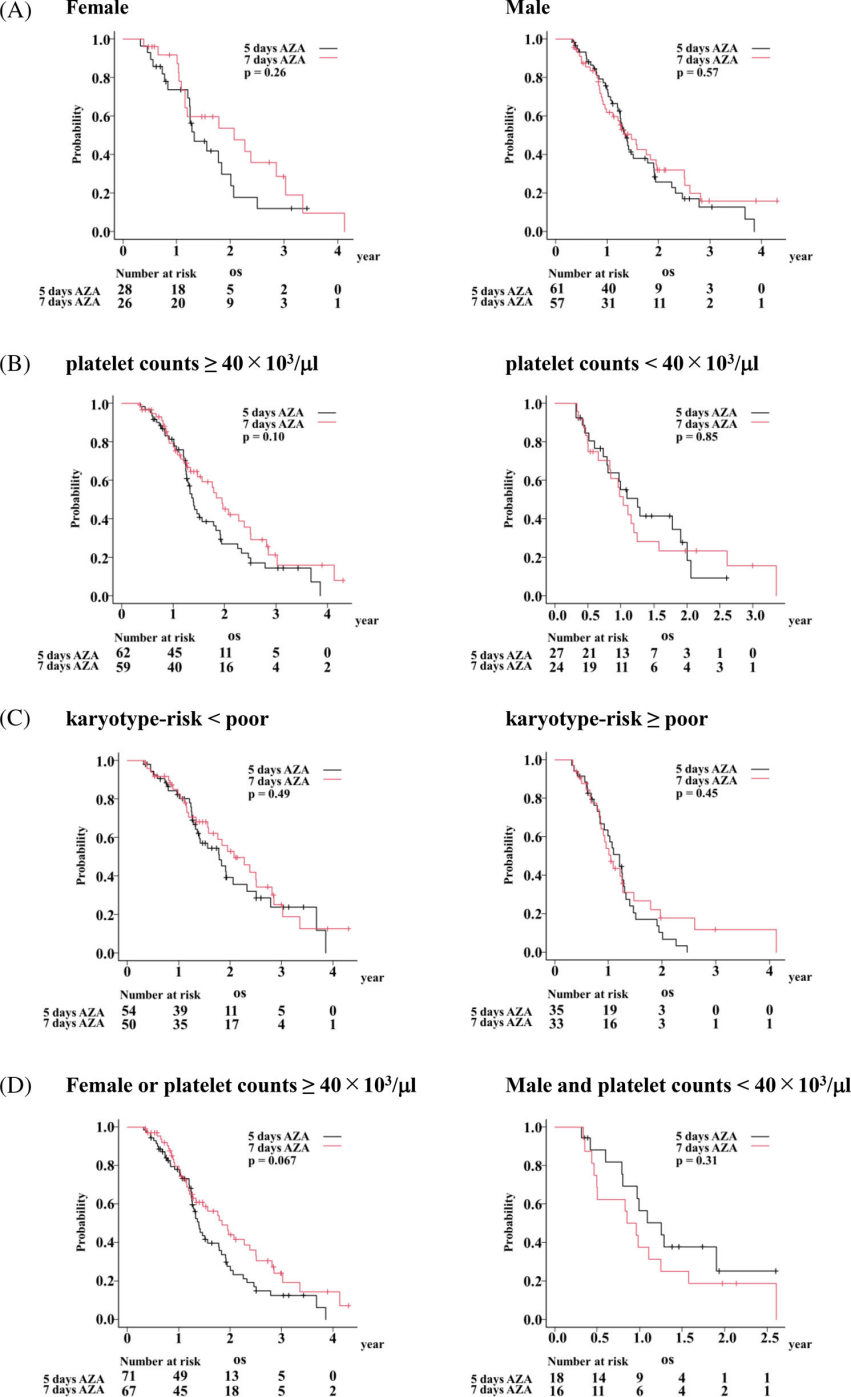
Subgroup analyses by sex, platelet counts, or karyotype in the validation cohort. Female and male (A), platelet counts <40 × 10^3^/μl and ≥40 × 10^3^/μl (B), karyotype‐risk <poor and ≥poor (C), and the union of female and platelet counts ≥40 × 10^3^/μl, and patients other than the union (D).

To validate the results of our retrospective cohort, the OS of 172 patients who were prospectively treated with 7‐ and 5‐day AZA (which correlates to the standard‐and the reduced‐doses, respectively, of the retrospective analysis) and survived 112 days or longer in the JALSG MDS212 study was compared.^18^ In this entire JALSG day 112 survivor cohort, OS was not significantly different between 7‐ and 5‐day AZA groups (HR, 0.80 [95% CI, 0.55–1.16]; *p* = .24). We then compared the OS between 7‐ and 5‐day AZA arms of the following three subcohorts: female patients, those with platelet counts ≥40 × 10^3^/μl, and those with karyotype‐risk ≥ poor. In the female and the platelet counts ≥40 × 10^3^/μl subcohorts, there was a tendency that OS was better in the 7‐day AZA arm (HR, 0.68 [95% CI 0.35–1.34], *p* = .26; and 0.69 [95% CI, 0.44–1.08], *p* = .10; respectively). These OS differences were not observed in the male patients and those with platelet counts <40 × 10^3^/μl. The difference between the two dose groups was marginal irrespective of karyotype‐risk in the JALSG day 112 survivor cohort (Table 4 and Figure 2). In the patients other than the male with platelet counts <40 × 10^3^/μl, there was a strong tendency that 7‐day AZA prolonged OS (Table 4 and Figure 2; *p* = .067).

**TABLE 4:** Subgroup analyses of overall survival in 172 patients from the JALSG MDS212 cohort.

	Dose of AZA	*N*	Median survival time (95% CI) (Day)	HR (95% CI)	*p* value
Female					.26
	5 days	28	484 (438–732)		
	7 days	26	756 (420–1104)	0.68 (0.35–1.34)	
Male					.57
	5 days	61	497 (443–652)		
	7 days	57	537 (349–710)	0.88 (0.56–1.37)	
Karyotype‐risk < poor					.49
	5 days	54	652 (483–848)		
	7 days	50	756 (569–1028)	0.84 (0.5–1.39)	
Karyotype‐risk ≥ poor					.45
	5 days	35	438 (307–474)		
	7 days	33	378 (309–463)	0.81 (0.47–1.4)	
Platelet counts <40 × 10^3^/μL					.85
	5 days	27	457 (288–695)		
	7 days	24	378 (238–455)	1.07 (0.55–2.07)	
Platelet counts ≥40 × 10^3^/μL					.10
	5 days	62	509 (458–652)		
	7 days	59	710 (489–911)	0.69 (0.44–1.08)	
Female or platelet counts ≥40 × 10^3^/μL					.067
	5 days	71	509 (458–652)		
	7 days	67	673 (463–868)	0.68 (0.44–1.03)	
Male and platelet counts <40 × 10^3^/μL					.31
	5 days	18	457 (288–NA)		
	7 days	16	329 (169–455)	1.52 (0.68–3.4)	

Abbreviations: 95% CI, 95% confidence interval; HR, hazard ratio; NA, not available.

Taken together, our results suggested that the standard‐dose AZA provided female patients and those with preserved platelet counts with better OS.

## 4 DISCUSSION

By a retrospective analysis of 151 MDS patients who survived 112 days or longer after the starting of AZA, we found that OS in the female and the platelet counts ≥40 × 10^3^/μl subcohorts significantly benefitted from the standard‐ rather than the reduced‐dose. In the cohort of the Phase III clinical trial comparing the 7‐ and 5‐day AZA scheduling,^18^ the OS tended to be better with 7‐day scheduling in the female patients and those with platelet counts ≥40 × 10^3^/μl.

In real‐world practice, either the standard‐ (7‐day) or the reduced‐dose (5‐day) regimen is chosen without prognostic stratification. Our results showed that both regimens may have equal efficiency for OS prolongation in the male MDS patients with platelet counts <40 × 10^3^/μl. On the other hand, the standard‐dose regimen reduced the risk of mortality by 57% and prolonged OS in the patients other than the male with platelet counts <40 × 10^3^/μl (Table 3 and Figure 1). According to these results, AZA is recommended to be administered as the standard‐dose, if the patients are not the male with platelet counts <40 × 10^3^/μl. This provides novel and crucial information for the physicians treating high‐risk MDS patients in choosing a treatment protocol, contributing to better quality of life and health economics.

A trend of shortened OS in patients receiving the reduced‐dose, compared to the standard‐dose, was previously described in a retrospective large cohort study.^25^ Such a trend was also described in a prospective study, albeit in a small number (*N* = 22) of patients based on a comparison with the AZA‐001 study.^9^ In a Phase III, JALSG MDS212 trial comparing 7‐ and 5‐day AZA for RAEB and RAEB‐t, although prematurely terminated because of poor recruitment, the 7‐day protocol showed a statistically insignificant but visible trend of better OS (MST 538 [95% CI, 396–711] days) than the 5‐day protocol (MST 477 [95% CI, 456–554] days).^18^ Failure to demonstrate statistical OS differences in that study was attributed by the authors to insufficient statistical power, given that time to leukemia trasnformation was significantly longer with the 7‐day protocol by multivariate analysis if only the centrally reviewed patients were investigated.^18^

In other previous reports comparing 5‐ and 7‐day protocols, the conclusions have been controversial. Laribi et al. introduced relative dose intensity (RDI) of AZA (the relative dose intensity is the percentage of the dose received by the patient on the dose that theoretically should have been administered) to investigate how the dose of AZA influence the outcome of 93 high‐risk MDS patients retrospectively. The OS and PFS were not different significantly with or without <80% RDI. The patients who responded to AZA were retrospectively divided into two groups; one group with <80% RDI and the other group without <80% RDI at the time when response was achieved. Dose reduction after the response was not considered. Then, they concluded that the group without <80% RDI showed significantly longer OS than the group with <80% RDI.^8^ The time of response could be approximated by the day112,^20^, ^21^ and the doses administered to standard‐dose group patients in our analysis resembled the group without <80% RDI in the report by Laribi, et al. Thus, the result of univariate analysis, but not multivariate analysis, in the current study may recapitulate the conclusion of Laribi et al. García‐Delgado et al. retrospectively compared three regimens, 5 days (AZA5), 7 days including 2‐day break (AZA 5‐2‐2), and 7 days (AZA7) in 200 patients with both high‐ and low‐risk MDS patients, with majority with the latter. In this analysis, AZA 5‐2‐2 had significantly better response rate than AZA 5 or AZA 7, but the OS was not different among three regimens.^11^ Fujimaki et al. compared the HI rate of their high‐risk MDS patients on the 5‐day AZA protocol with the HI rate of the high‐risk MDS patients on the 7‐day AZA protocol, and concluded that the HI rate was similar in both protocol.^10^

In the present study, 32 patients who died sooner than 112 days were removed from the landmark analysis for the Survivors112, but this removal could affect the conclusion. We explored potential differences between these short survivors and the Survivors112 by comparing the characteristics of patients. Although information on performance status and comorbidities was missing, we did not detect significant differences in other characteristics of patients between the two groups such as age, sex, diagnosis, risks on IPSS‐R and karyotype, hematological parameters at the first administration of AZA, and bone marrow blast percentage at diagnosis or within 3 months before AZA start (Table S1).

In the Survivors112, the response to AZA by standard‐dose was significantly better than reduced‐dose in univariate and multivariate analyses. OS differences within Survivors112 were found between the two cumulative AZA dose groups in the univariate analysis. However, multivariate analysis did not show a significant difference. This could be due to biases or the dilution effect, which obscured findings in a specific subgroup of patients by other patients, according to the results of subgroup analyses in our study.

We reported that the standard‐dose improved OS in female and the platelet counts ≥40 × 10^3^/μl subcohorts within Survivors112. While we found an association between standard‐dose and longer OS in specific subcohorts, there might be factors that influenced the results other than the standard‐dose, given that the nature of retrospective analysis.

Preserved platelet count is an important component for prognosis prediction in IPSS‐R^23^ and, thus, should be selective for a subcohort with better OS. While this could be correlated to tolerability, clear explanations on why platelet, but not other blood cell lineages, affect the AZA dose preference remain elusive.

It was unexpected that the OS advantage imparted by the standard‐dose was seen in female but not male patients. The activity of cytidine deaminase that inactivates AZA is known to be lower in females than males in a murine model.^26^ Likewise, as cytarabine clearance from blood is known to be faster in males than females (as reported in a clinical trial),^27^ AZA metabolism could differ between females and males and activity could persist in females if the dose is the same. It is, however, unclear whether and how this knowledge can explain the differences in observed outcomes.

There were several limitations in our study. First, the patients of our cohort came from 5 hospitals in Ibaraki prefecture, Japan, thus, there might be a geographical bias. Second, performance status and comorbidities were missing in our study. Third, the cumulative dose of AZA was surrogate index of 5‐day dose or 7‐day dose, but not equal to those. Fourth, the inclusion criteria between the current retrospective study and the JALSG MDS212 study were different. Of note, 26.8% of patients in the current retrospective cohort were MDS with low blasts, in contrast to the JALSG MDS212 cohort that included only RAEB and RAEB‐t patients. Fifth, availability of the mutation profiles was incomplete in our retrospective cohort and not useful for the analysis.

Prospective study including large numbers of MDS patients is ideal to confirm results from our retrospective cohort. Given the premature termination of JALSG MDS212 prospective study due to poor recruitment, however, it might not be easy to perform a new prospective study comparing AZA doses in the future when new drugs would be equipped. In another way, better‐designed retrospective analysis which takes the limitations of our cohort into account may be feasible. Simultaneously, it is warranted to elucidate the mechanism how the gender and platelet count influence OS under different AZA doses.

In conclusion, we identified by retrospective analysis that female and platelet counts ≥40 × 10^3^/μl subcohorts of MDS (including oligoblastic AML), receive OS benefits from standard‐dose rather than reduced‐dose AZA. The same tendency was observed in the validation cohort independent of our cohort, although statistical significance was not seen.

## AUTHOR CONTRIBUTIONS

**Shinichi Ogawa:** Conceptualization (lead); data curation (lead); formal analysis (lead); investigation (lead); methodology (lead); resources (lead); writing – original draft (lead). **Tatsuhiro Sakamoto:** Conceptualization (equal); data curation (equal); formal analysis (equal); methodology (equal); resources (equal). **Ryota Matsuoka:** Formal analysis (equal). **Kantaro Ishitsuka:** Formal analysis (supporting). **Yasuko Ogino:** Resources (equal). **Ayano Sootome:** Resources (equal). **Kenichi Makishima:** Formal analysis (supporting). **Chikashi Yoshida:** Resources (equal). **Yufu Ito:** Resources (equal). **Seiichi Shimizu:** Resources (equal). **Takuya Suyama:** Resources (equal). **Atsushi Shinagawa:** Resources (equal). **Takayoshi Ito:** Resources (equal). **Naoshi Obara:** Validation (equal). **Manabu Kusakabe:** Resources (equal). **Mamiko Sakata‐Yanagimoto:** Validation (equal). **Yasushi Miyazaki:** Validation (equal). **Yasuhito Nannya:** Validation (lead). **Shigeru Chiba:** Supervision (lead); writing – review and editing (lead).

## CONFLICT OF INTEREST STATEMENT

S.Ogawa received honorarium from Novartis, Janssen Pharmaceutical, Nippon Shinyaku, Chugai Pharmaceutical, Daiichi Sankyo, and Bristol‐Myers Squibb outside the submitted work. The husband of A. Sootome has stock of Otsuka Holdings. K.Makishima received honorarium from Ono Pharmaceutical outside the submitted work. C.Yoshida received honorarium from Novartis, Otsuka Pharmaceutical, AbbVie GK, Janssen Pharmaceutical, Nippon Shinyaku, Chugai Pharmaceutical., and honoraria and research funding from Bristol‐Myers Squibb outside the submitted work. Y. Ito received honorarium from Nippon Shinyaku outside the work. T. Suyama received honorarium from Abbvie, Meiji‐Seika, Sanofi, Janssen Pharmaceutical, Kyowa‐Kirin and Nippon Shinyaku outside the work, and has stock of Takeda Pharmaceutical. T. Ito received honorarium from Janssen Pharmaceutical, Ono Pharmaceutical, Nippon Shinyaku, Chugai Pharmaceutical, Sanofi, Meiji‐Seika, Bristol‐Myers Squibb, Takeda Pharmaceutical, Novartis, and Otsuka Pharmaceutical outside the work. N.Obara received research funding from Kyowa‐Kirin outside the submitted work. M.Kusakabe received honorarium from Janssen Pharmaceutical, Astellas, Takeda Pharmaceutical, Chugai Pharmaceutical, Astrazeneca, Meiji‐Seika outside the submitted work. M.Sakata‐Yanagimoto received research funding from Eisai, Bristol‐Myers Squibb, Otsuka and Mundipharma outside the submitted work, and honorarium from Nippon Shinyaku, Kyowa‐Kirin, Chugai Pharmaceutical, Astellas, Meiji‐Seika, Zenyaku Kogyo, Mundipharma outside the submitted work. Y.Miyazaki received honorarium from Nippon‐Shinyaku, Bristol‐Myers Squibb, Novartis, Sumitomo Pharma, Kyowa‐Kirin, Abbvie, Daiichi‐Sankyo, Takeda Pharmaceutical, Janssen Pharmaceutical, Astellas, Pfizer, Chugai Pharmaceutical, SymBio, Otsuka Pharmaceutical, and Research funding from Sumitomo‐Dainippon outside the submitted work. Y.Nannya received honorarium from Takeda Pharmaceutical, Pfizer, Chugai Pharmaceutical, Sumitomo Pharma, Astrazeneca, Kyowa‐Kirin, Asahi Kasei Pharma, Nippon Shinyaku, Fuji Pharma, Janssen Pharmaceutical, Filgen, Otsuka Pharmaceutical and Bristol Myers Squibb outside the submitted work, and research funding from Daiichi Sankyo RD Novare outside the submitted work. Y. Nannya participates in advisory boards in Novartis and Otsuka Pharmaceutical outside the submitted work. S.Chiba reports research funding from Ono Pharmaceutical, Chugai Pharmaceutical, Eisai, Kyowa Kirin, Astellas, Bayer, and Thyas outside the submitted work, and honorarium from Nippon Shinyaku, Chugai Pharmaceutical, Eisai, Kyowa‐Kirin, Astellas, Janssen Pharmaceutical, Asahi Kasei, Sanofi, Meiji‐seika, Takeda Pharmaceutical, AMGEN, Ono Pharmaceutical, MSD, Astrazeneca, Abbvie, Daiichisankyo, Pfizer, and Otsuka Pharmaceutical outside the submitted work. Any of the authors of this manuscript is not a current Editor or Editorial Board Member of Cancer Science. All authors had full access to all the data in this study and had final responsibility for the decision to submit for publication.

## ETHICS STATEMENT

Approval of research protocol by an institutional review board. This retrospective study was approved by the institutional review board in each hospital.

## INFORMED CONSENT

N/A. This retrospective study was based on the medical records. Obeying the approval of each institutional review board, we performed opt‐out in each hospital instead of written informed consent.

## Supporting information


**Table S1.** Patient characteristics.
**Table S2.** Univariate and multivariate analyses of hematological improvement from AZA.
**Table S3.** Univariate and multivariate analyses of overall survival.
**Table S4.** Patient characteristics divided by cumulative dose of AZA at day 112 in the propensity score‐matched analysis.
**Figure S1.** Box plot of AZA cumulative dose at day 112 for the Survivors112 cohort.
**Figure S2.** Overall survivals in the Survivors112 cohort. Comparison between patients with (a) karyotype risks, (b) IPSS‐R risks, (c) hemoglobin levels (Hb), and (d) cumulative AZA doses at day 112.
**Figure S3.** Distribution of propensity scores. Histogram of propensity scores before 1:1 caliper matching (caliper 0.2) (a), histogram of propensity scores after 1:1 caliper matching (caliper 0.2) (b), distribution of propensity scores depicted by Kernel density estimation (c).

## Data Availability

The raw data that support the findings of this study are available from the corresponding author upon reasonable request.
