# Supplementary material for: Female and preserved platelet count subgroups of myelodysplastic syndrome patients benefit from standard‐dose azacitidine
Source: Cancer Rep (Hoboken). 2023 Nov 28;7(1):e1938. doi: 10.1002/cnr2.1938 (PMC10809187; doi:10.1002/cnr2.1938)
Supplement: Supplementary file 1 — Table S1. Patient characteristics. Table S2. Univariate and multivariate analyses of hematological improvement from AZA. Table S3. Univariate and multivariate analyses of overall survival. Table S4. Patient characteristics divided by cumulative dose of AZA at day 112 in the propensity score‐matched analysis. Figure S1. Box plot of AZA cumulative dose at day 112 for the Survivors112 cohort. Figure S2. Overall survivals in the Survivors112 cohort. Comparison between patients with (a) karyotype risks, (b) IPSS‐R risks, (c) hemoglobin levels (Hb), and (d) cumulative AZA doses at day 112. Figure S3. Distribution of propensity scores. Histogram of propensity scores before 1:1 caliper matching (caliper 0.2) (a), histogram of propensity scores after 1:1 caliper matching (caliper 0.2) (b), distribution of propensity scores depicted by Kernel density estimation (c). [file CNR2-7-e1938-s001.pdf]

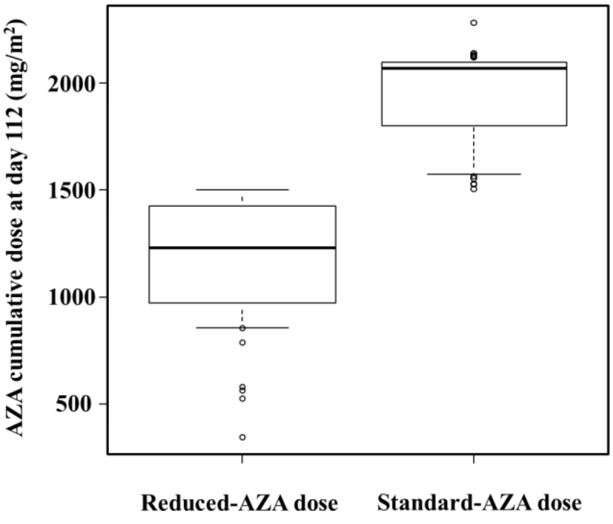

Supporting Figure 1

Box plot of AZA cumulative dose at day 112 for the Survivors112 cohort.

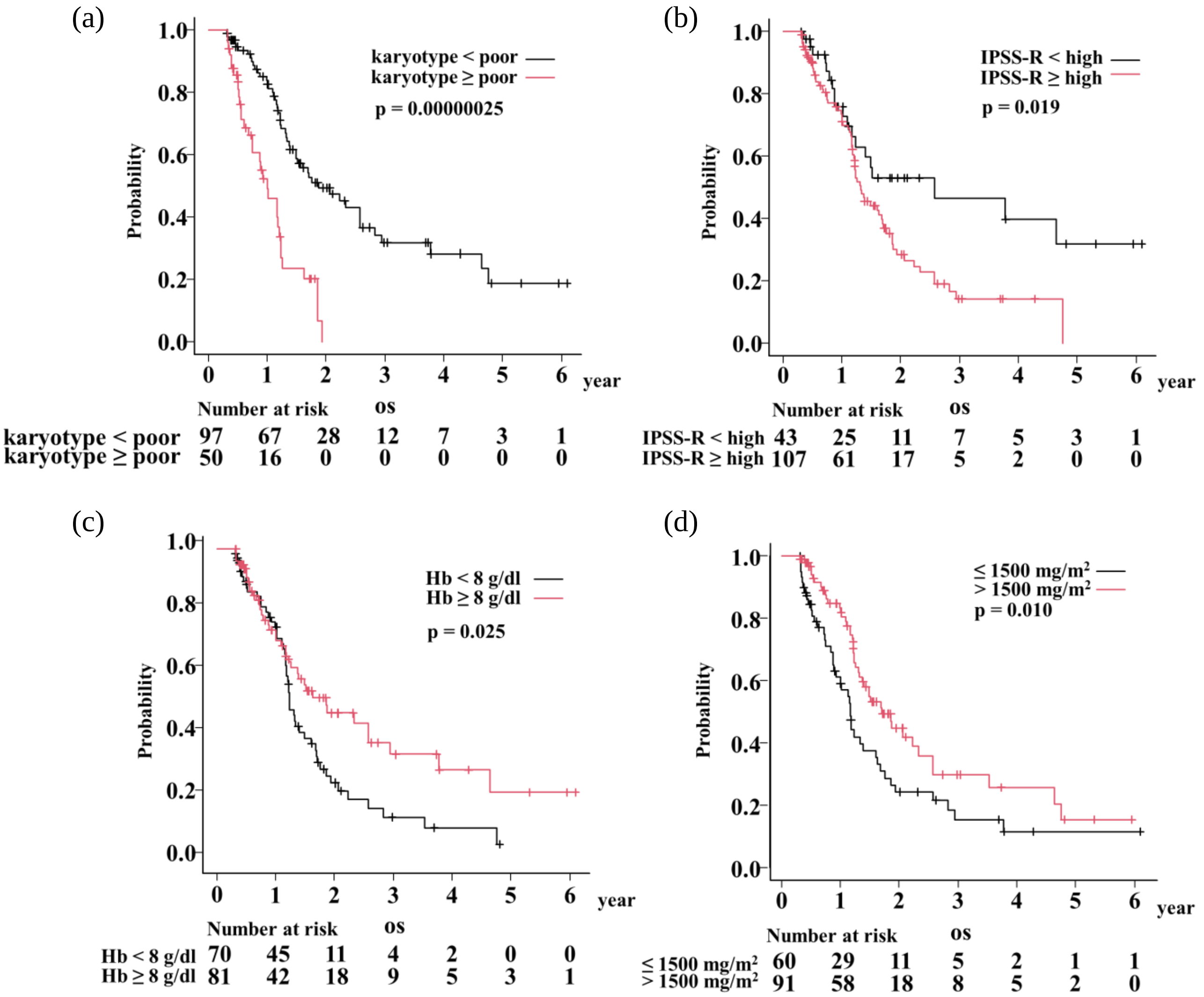

Supporting Figure 2

Overall survivals in the Survivors112 cohort. Comparison between patients with (a) karyotype risks, (b) IPSS-R risk, (c) hemoglobin levels (Hb), and (d) cumulative AZA doses at day 112.

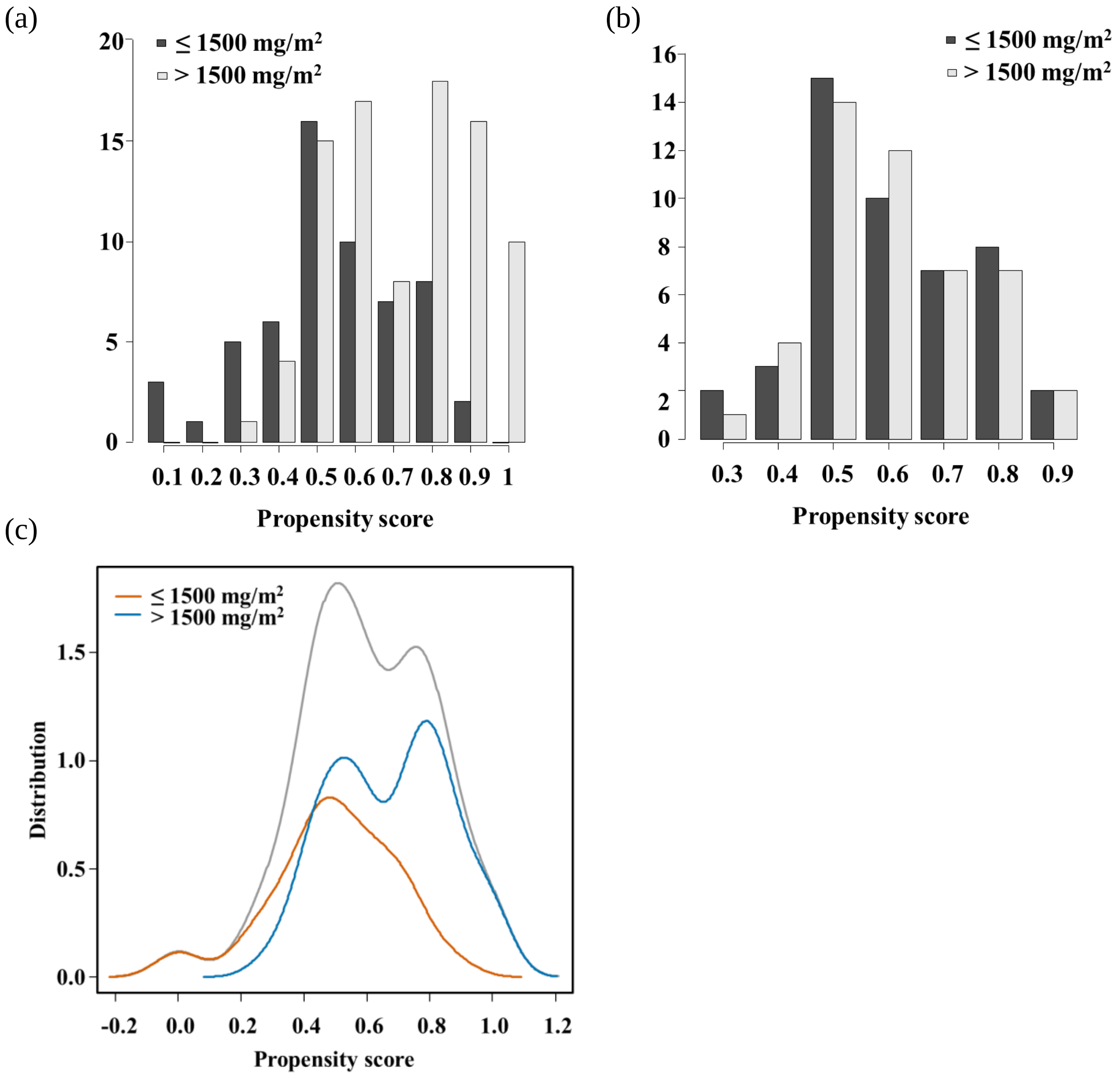

Supporting Figure 3

Distribution of propensity scores. Histogram of propensity scores before 1:1 caliper matching (caliper 0.2) (a), histogram of propensity scores after 1:1 caliper matching (caliper 0.2) (b), distribution of propensity scores depicted by Kernel density estimation (c).

Supporting Table 1 Patient characteristics

	All Patients (%)	OS < 112 days	OS ≥ 112 days	P Value
N	183	32	151	
Age, median [range]	72 [29, 90]	69 [40, 86]	72 [29, 90]	0.30
Sex				0.84
Male	123 (67.2)	21 (65.6)	102 (67.5)	
Female	60 (32.8)	11 (34.4)	49 (32.5)	
WHO 2016 criteria				0.67
MDS-SLD	5 (2.7)	1 (3.1)	4 (2.6)	
MDS-MLD	35 (19.1)	8 (25.0)	27 (17.9)	
MDS-EB1	58 (31.7)	8 (25.0)	50 (33.1)	
MDS-EB2	57 (31.1)	11 (34.4)	46 (30.5)	
AML-MRC	9 (10.4)	2 (6.2)	17 (11.3)	
MDS with isolated del(5q)	1 (0.5)	0 (0.0)	1 (0.7)	
MDS-RS	4 (2.2)	2 (6.2)	2 (1.3)	
CMML	1 (0.5)	0 (0.0)	1 (0.7)	
tMN	2 (1.1)	0 (0.0)	2 (1.3)	
MDS-U	1 (0.5)	0 (0.0)	1 (0.7)	
IPSS-R risk group				0.13
Very low	2 (1.1)	0 (0.0)	2 (1.3)	
Low	19 (10.4)	3 (9.4)	16 (10.6)	
Intermediate	30 (16.4)	4 (12.5)	26 (17.2)	
High	55 (30.1)	5 (15.6)	50 (33.1)	
Very high	75 (41.0)	19 (59.4)	56 (37.1)	
NA	2 (1.1)	1 (3.1)	1 (0.7)	
IPSS-R karyotype group				0.59
Very good	3 (1.6)	0 (0.0)	3 (2.0)	
Good	70 (38.3)	9 (28.1)	61 (40.4)	
Intermediate	40 (21.9)	7 (21.9)	33 (21.9)	
Poor	13 (7.1)	2 (6.2)	11 (7.3)	
Very poor	52 (28.4)	13 (40.6)	39 (25.8)	
NA	5 (2.7)	1 (3.1)	4 (2.6)	
Bone marrow blast %, median [range]	7.5[0.0, 29.8]	5.2 [0.2, 29.2]	7.6 [0.0, 29.8]	0.17
Neutrophile count (/μL), median [range]	912 [47, 22243]	1161 [60, 7040]	888 [47, 22243]	0.36
Hemoglobin (g/dL), median [range]	8.1 [2.4, 12.9]	7.5 [3.4, 12.0]	8.1 [2.4, 12.9]	0.23
Platele count (× 10^3/μL), median [range]	62 [4, 629]	57 [4, 380]	63 [5, 629]	0.54
Azacitidine course, median [range]	6 [1, 61]			
Median follow up time, days (95% CI)	335 (262 - 422)			
Median survival time, days (95% CI)	479 (428 - 594)			
OS at 112 days , % (95% CI)	91.5 (86.4 - 94.8)			
OS at 1 year, % (95% CI)	66.6 (58.3 - 73.6)			
OS at 2 year, % (95% CI)	32.8 (24.5 - 41.4)			

Abbreviations: MDS-SLD, myelodysplastic syndrome with single lineage dysplasia;
MDS-MLD, myelodysplastic syndrome with multilineage dysplasia;
MDS-EB1, myelodysplastic syndrome with excess blasts 1;
MDS-EB2, myelodysplastic syndrome with excess blasts 2;
AML-MRC; acute myeloid leukemia with myelodysplasia-related changes;
MDS-RS, myelodysplastic syndrome with ring sideroblasts;
CMML, chronic myelomonocytic leukemia;
tMN, therapy related myeloid neoplasms; MDS-U, myelodysplastic syndrome, unclassifieable;
IPSS-R, revised international prognostic scoring system; NA, not available;
95% CI, 95% confidence interval; OS, overall survival

Supporting Table 2 Univariate and multivariate analyses of hematological improvement from AZA

	Univariate Analyses				Multivariate Analysis	
	N	No Response (%)	Response (%)	P Value	Odds Ratio (95% CI)	P Value
Age				0.73		0.37
Age < 75	91	43 (47.3)	48 (52.7)			
Age ≥ 75	55	24 (43.7)	31 (56.3)		1.43 (0.66 - 3.12)	
Sex				0.049		0.044
Male	100	40 (40.0)	60 (60.0)		2.34 (1.02 - 5.33)	
Female	46	27 (58.7)	19 (41.3)			
IPSS-R				0.86		
Very low, low or intermediate	43	19 (44.2)	24 (55.8)			
High or very high	102	48 (47.1)	54 (52.9)			
Karyotype (IPSS-R criteria)				0.079		0.24
Very good, good or intermediate	94	38 (40.4)	56 (59.6)			
Poor or very poor	48	27 (56.4)	21 (43.6)		0.63 (0.29 - 1.37)	
Bone marrow blast percentage				0.17		0.13
< 10%	92	38 (41.3)	54 (58.7)			
≥ 10%	54	29 (53.8)	25 (46.2)		0.54 (0.24 - 1.19)	
Neutrophile count (/μL)				0.091		0.10
< 800 /μL	39	13 (33.3)	26 (66.7)			
≥ 800 /μL	107	54 (50.5)	53 (49.5)		0.49 (0.21 - 1.15)	
Hemoglobin (g/dl)				0.41		0.71
< 8 g/dL	66	33 (50.0)	33 (50.0)			
≥ 8 g/dL	80	34 (42.5)	46 (57.5)		1.16 (0.53 - 2.53)	
Platelet count ($\times 10^3$/μL)				0.16		0.24
< 40 $\times 10^3$/μL	46	17 (37.0)	29 (63.0)			
≥ 40 $\times 10^3$/μL	100	50 (50.0)	50 (50.0)		0.61 (0.26 - 1.40)	
Cumulative dose of AZA at day 112				0.00064		0.00044
≤ 1500 mg/m^2 (reduced dose of AZA)	58	37 (63.8)	21 (36.2)			
> 1500 mg/m^2 (standard dose of AZA)	88	30 (34.1)	58 (65.9)		4.13 (1.87 - 9.10)	

Abbreviations: IPSS-R, revised prognostic scoring system; AZA, azacitidine; 95% CI, 95% confidence interval.

Supporting Table 3 Univariate and multivariate analyses of overall survival

	Univariate Analyses of Overall Survival				Multivariate Analysis of Overall Survival	
	N	Median Survival Time (95% CI) (Day)	HR (95% CI)	P Value	HR (95% CI)	P Value
Total						
Age				0.29		0.14
age < 75	94	543 (448 - 682)				
age ≥ 75	57	458 (422 - 623)	1.26 (0.82 - 1.93)		1.46 (0.89 - 2.41)	
Sex				0.75		0.53
Male	102	499 (438 - 640)	1.08 (0.68 - 1.71)		1.19 (0.69 - 2.03)	
Female	49	584 (412 - 940)				
IPSS-R				0.019		
Very low, low or intermediate	43	938 (427 - NA)				
High or very high	107	482 (429 - 617)	1.85 (1.10 - 3.12)			
Karyotype				0.00000025		0.000012
Very good, good or intermediate	97	682 (505 - 940)				
Poor or very poor	50	364 (271 - 438)	3.29 (2.04 - 5.31)		3.14 (1.88 - 5.23)	
Bone marrow blast percentage				0.30		0.62
< 10%	94	543 (438 - 707)				
≥ 10%	57	484 (426 - 623)	1.26 (0.82 - 1.94)		1.13 (0.70 - 1.81)	
Neutrophile count (/μL)				0.77		0.35
< 800 /μL	42	542 (443 - 751)				
≥ 800 /μL	109	509 (427 - 676)	0.93 (0.58 - 1.51)		0.77 (0.45 - 1.33)	
Hemoglobin (g/dl)				0.025		0.053
< 8 g/dL	70	450 (427 - 584)				
≥ 8 g/dL	81	676 (458 - 1072)	0.62 (0.40 - 0.95)		0.61 (0.36 - 1.01)	
Platelet count (× 10^3/μL)				0.33		0.88
< 40 × 10^3/μL	47	611 (428 - 812)				
≥ 40 × 10^3/μL	104	505 (445 - 682)	1.26 (0.79 - 2.0)		1.04 (0.60 - 1.80)	
Cumulative dose of AZA at day 112				0.010		0.28
≤ 1500 mg/m^2 (reduced dose of AZA)	60	427 (321 - 584)				
> 1500 mg/m^2 (standard dose of AZA)	91	623 (482 - 850)	0.58 (0.38 - 0.88)		0.76 (0.47 - 1.25)	
Response				0.00025		0.016
Gain of hematological improvement	79	751 (617 - 1072)				
No gain of hematological improvement	67	422 (335 - 479)	2.21 (1.43 - 3.42)		1.89 (1.13 - 3.18)	

Abbreviations: IPSS-R, revised prognostic scoring system; AZA, azacitidine; 95% CI, 95% confidence interval; HR, hazard ratio.

Supporting table 4 Patient characteristics divided by cumulative dose of AZA at day 112 in the propensity score-matched analysis

	Cumultive dose of AZA at day 112			
	≤ 1500 mg/m^2 (%)	> 1500 mg/m^2 (%)	P Value	SMD
N	47	47		
Age, median [range]	74 [29, 90]	71 [42, 86]	0.32	0.0060
Sex			1	0.047
Male	34 (72.3)	33 (70.2)		
Female	13 (27.7)	14 (29.8)		
WHO 2016 criteria			0.94	0.18
MDS-SLD	1 (2.1)	1 (2.1)		
MDS-MLD	10 (21.3)	11 (23.4)		
MDS-EB1	17 (36.2)	20 (42.6)		
MDS-EB2	14 (29.8)	11 (23.4)		
AML-MRC	5 (10.6)	4 (8.5)		
MDS with isolated del(5q)	0 (0.0)	0 (0.0)		
MDS-RS	0 (0.0)	0 (0.0)		
CMML	0 (0.0)	0 (0.0)		
tMN	0 (0.0)	0 (0.0)		
MDS-U	0 (0.0)	0 (0.0)		
IPSS-R risk group			0.18	0.47
Very Low	0 (0.0)	0 (0.0)		
Low	5 (10.6)	4 (8.5)		
Intermediate	5 (10.6)	12 (25.5)		
High	17 (36.2)	10 (21.3)		
Very high	20 (42.6)	21 (44.7)		
IPSS-R karyotype group			1	0.078
Very good	0 (0.0)	0 (0.0)		
Good	15 (31.9)	16 (34.0)		
Intermediate	12 (25.5)	12 (25.5)		
Poor	5 (10.6)	4 (8.5)		
Very poor	15 (31.9)	15 (31.9)		
Transplantation			0.74	0.14
No	43 (91.5)	41 (87.2)		
Yes	4 (8.5)	6 (12.8)		
Bone marrow blast %, median [range]	7.0 [0.40, 26.5]	7.0 [0.0, 27.6]	0.48	0.16
Neutrophile count (/μL), median [range]	903 [110, 22243]	937 [110, 19757]	0.37	0.11
Hemoglobin (g/dL), median [range]	7.8 [4.2, 12.9]	7.7 [2.4, 12.9]	0.60	0.12
Platelet count (× 10^3/μL), median [range]	61 [10, 230]	70 [8, 364]	0.97	0.12

Patients were included whose overall survival was 112 days or longer.

Abbreviations: MDS-SLD, myelodysplastic syndrome with single lineage dysplasia; MDS-MLD, myelodysplastic syndrome with multilinea
MDS-EB1, myelodysplastic syndrome with excess blasts 1; MDS-EB2, myelodysplastic syndrome with excess blasts 2;
AML-MRC, acute myeloid leukemia with myelodysplasia-related changes; MDS-RS, myelodysplastic syndrome with ring sideroblasts;
CMML, chronic myelomonocytic leukemia; tMN, therapy related myeloid neoplasms; MDS-U, myelodysplastic syndrome, unclassifieable;
IPSS-R, revised international prognostic scoring system; SMD, standardized mean difference;
